# Artery-Only Ear Replantation in a Child: A Case Report With Daily Photographic Documentation

**Published:** 2016-12-28

**Authors:** Shaun D. Mendenhall, Justin D. Sawyer, Joshua M. Adkinson

**Affiliations:** ^a^The Institute for Plastic Surgery, Southern Illinois University School of Medicine, Springfield; ^b^Division of Plastic Surgery, Department of Surgery, Indiana University School of Medicine, Indianapolis

**Keywords:** ear replantation, leech therapy, venous congestion, microsurgery, artery-only replantation

## Abstract

**Objective:** Ear replantation poses a significant technical challenge even for the skilled microsurgeon. Many ear amputations result from avulsion and thus have damaged and often diminutive vessels with a paucity of veins. Artery-only replantation is an option for ear salvage, but little is published on the clinical course and appearance after this procedure. **Methods:** A subtotal ear replantation was performed on a 10-year-old boy without a venous anastomosis. Leech therapy was used to manage venous congestion postoperatively, and daily photography was performed to document the clinical course. **Results:** Postoperative venous congestion was successfully managed with leech therapy. Four days after the replantation, arterial thrombosis occurred that required a take back and salvage with an interposition vein graft for arterial repair. Native venous drainage and arterial revascularization from skin edges were evident by postoperative day 12, and leeches were discontinued on day 14. The patient required debridement of the posterior ear and superior helix necrotic skin, with burying of the upper portion of the ear in a superior auricular skin flap. The ear was subsequently released from the head, and the exposed portions were covered successfully with a full-thickness skin graft. **Conclusions:** While arterial and venous anastomoses should always be attempted, arterial-only ear replantation can provide excellent results when venous congestion is properly managed. Daily photography can be a useful tool to monitor subtle skin color changes that may indicate native venous drainage and arterial revascularization.

Ear replantation is a technically challenging procedure that, when successful, can fully restore aesthetic and functional characteristics of the ear. The success rate of ear replantation has vastly increased in the microsurgical era since the first successful microvascular replantation in 1980.^1^ The procedure is often complicated by the small caliber of pertinent vasculature, the frequent avulsion nature of injury, and the confined space in which to operate in the retroauricular sulcus. While it is seems ideal to reestablish arterial and venous blood flow in ear replantation, venous anastomoses are often difficult and may not be possible. We present the case of a 10-year-old boy with a subtotal ear amputation from a dog bite who underwent arterial-only replantation with postoperative leech therapy to manage venous congestion. We performed extensive photographic documentation of the clinical course and revascularization of the replanted ear.

## METHODS

### Replantation

**Figure 1:**
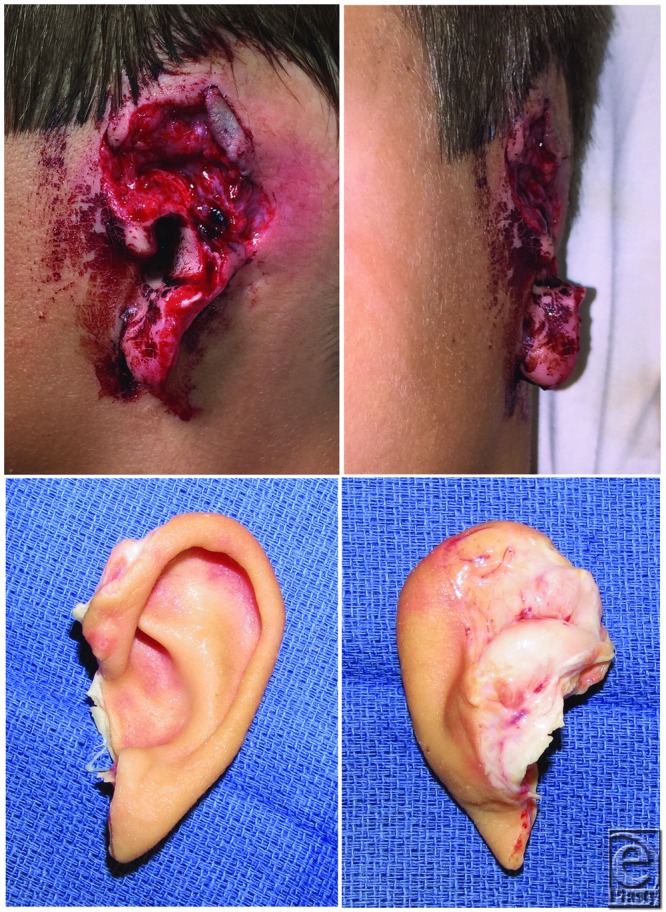
Amputation site with only the medial portion of lobule and tragus remaining (top) and the amputated ear prior to replantation (bottom).

**Figure 2:**
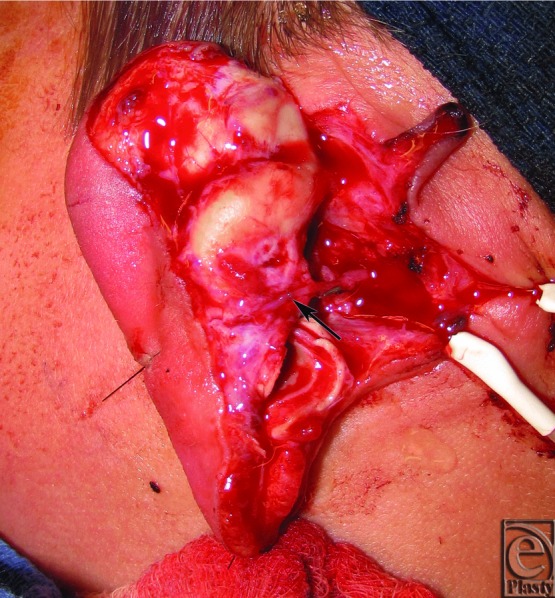
Replanted ear reflected anteriorly immediately after reperfusion. The vascular pedicle can be seen approximately half way up the ear (arrow) based on a small branch of the posterior auricular artery.

**Figure 3:**
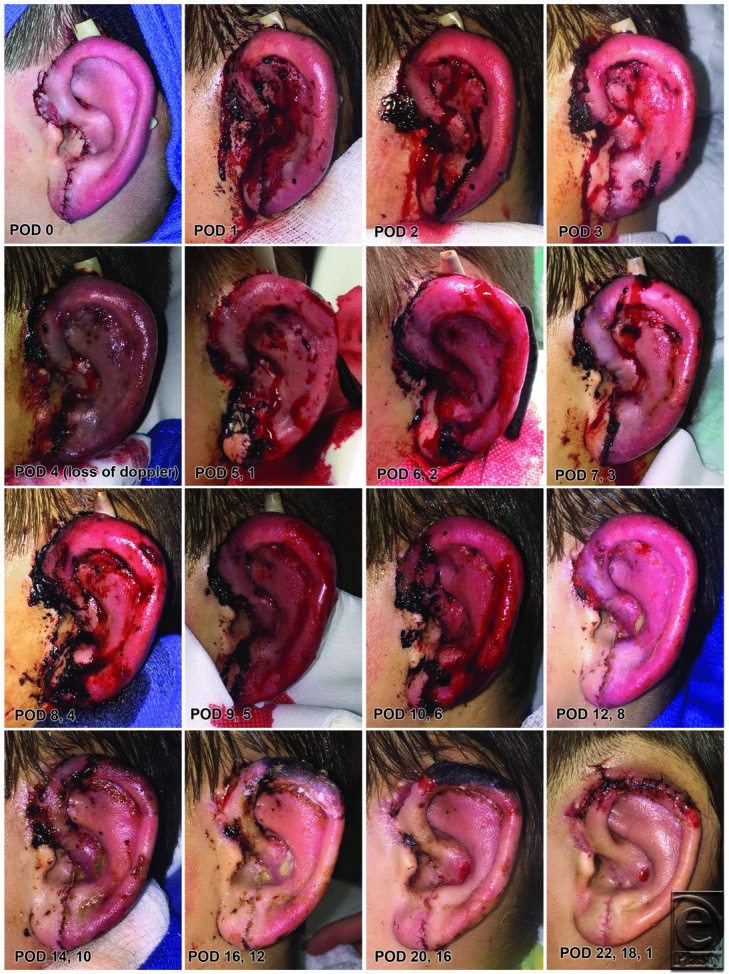
Day-by-day progression of the replanted ear throughout the early postoperative course from POD 0 to 22. Note the acute color change due to arterial thrombosis on POD 4 that required take back to the operating room for salvage with a vein graft. Also note the dramatic color change on POD 12, 8 associated with return of native venous drainage and arterial neovascularization progressing from the lobule upward. POD indicates postoperative day.

A healthy 10-year-old boy suffered a dog bite avulsion of the left ear, leaving only the medial portion of the lobule and the tragus behind (Fig 1). He presented approximately 2 hours after the injury as a transfer from an outside hospital. Prior to the patient's arrival to the operating room (OR), the amputated ear was soaked in betadine solution and prepped for replantation. Under microscopic exploration, one 0.8-mm arterial branch was identified on the posterior surface of the ear, but no suitable vein was found. The patient was placed under general anesthesia, the wounds were copiously irrigated with saline, and devitalized tissue was judiciously debrided. The anterior ear was partially inset to prevent movement during microsurgical repair. The artery was anastomosed to a branch of the posterior auricular artery. Following removal of the arterial clamps, the ear immediately reperfused (Fig 2). No adequate vein was found after reperfusion. As such, he was placed on a therapeutic heparin drip, and a 1/4-in Penrose drain was placed for fluid egress in preparation for future leech therapy. The cartilage and the skin were loosely reapproximated with absorbable suture (Fig 3, postoperative day [POD] 0). The incisions were cleaned and dressed, and the patient was sent to recovery. Total operative time was 4.5 hours.

### Leech therapy

**Figure 4:**
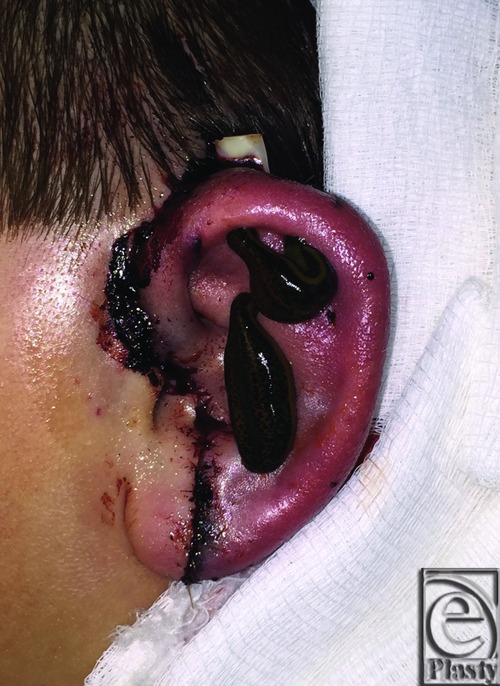
Application of leeches rapidly reduced venous congestion in the ear (postoperative day 0, hour 8).

**Figure 5:**
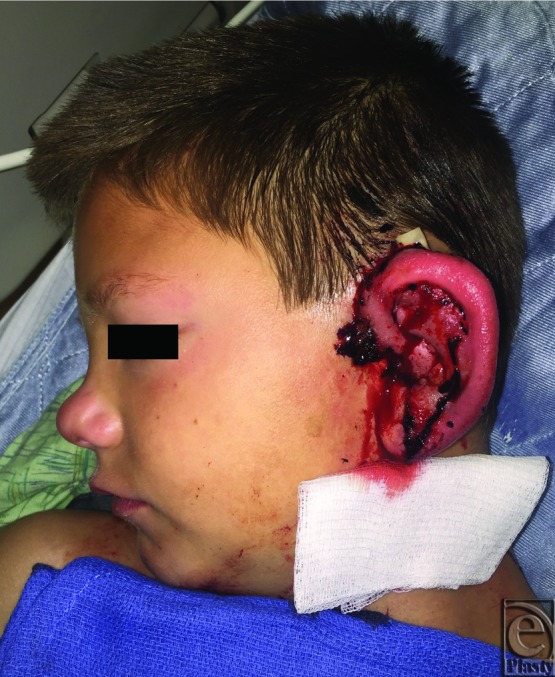
Extensive postoperative swelling is noted in this arterial-only total ear replant, peaking on postoperative day 2.

Leeches were ordered in an expedited fashion and arrived 8 hours after surgery (Leeches U.S.A. Ltd, Westbury, NY). Given the delay, the ear had become substantially venous congested (Fig 4). Leech therapy was initiated, with 2 leeches every 2 to 3 hours and gradually decreased as tolerated (Table 1). The patient remained on a heparin drip titrating to partial thromboplastin time of 60 to 85 for 7 days, daily aspirin 81 mg, and empiric antibiotics against the canine and leech flora for 3 weeks. The patient required a total of 6 units of transfused red blood cells during his leeching course. The patient experienced extensive swelling of the ear, which peaked on POD 2 (Fig 5).

### Take back to the OR

On POD 4, after 2 episodes of forceful vomiting, an acute color change was noted in the patient's ear and the Doppler signal was lost, indicating the loss of arterial perfusion (Fig 3, POD 4). The patient was urgently taken back to the OR and upon exploration of the posterior ear, while keeping the anterior ear suture line intact, a posterior ear hematoma was noted along with thrombosis of the previous arterial anastomosis. The anastomosis was resected, and an interpositional vein graft was used for arterial reconstruction. Satisfactory reperfusion of most of the ear was noted. However, a small portion at the root of the helix and the concha remained cyanotic. A small blister appeared on the concha postoperatively that progressed to an ulcer extending to cartilage. This was treated with topical mafenide acetate 8.5% cream twice daily until complete secondary healing.

## RESULTS

**Figure 6:**
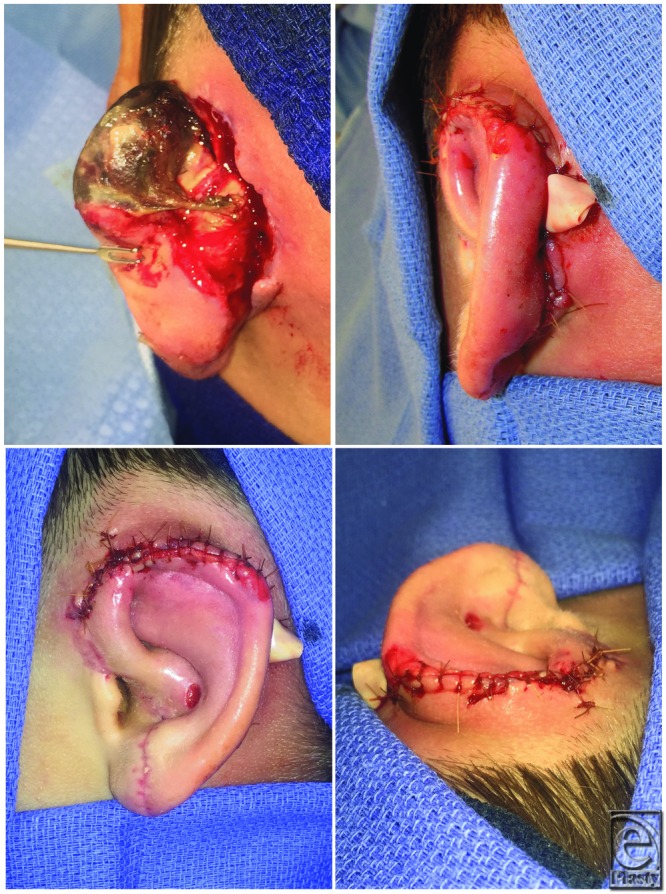
The patient had skin necrosis noted at the superior portion of the helix and postauricular skin, along with wound separation on the posterior portion of the ear. This was debrided, closed posteriorly, and the superior part of the ear was buried in a superior-auricular skin pocket (postoperative day 21, 17, 0).

Following revascularization, there was poor wound healing of the posterior aspect of the ear; this was managed with wet-to-dry dressings. The anterior aspect of the ear continued to improve in color and swelling, as native venous drainage and arterial flow progressed from the lobule upward (Fig 3, POD 12, 8). There was mild compromise of the superior helix toward the end of initial hospitalization. The patient was discharged on POD 14 after initial replantation, approximately 24 hours after complete weaning of the leeches. Later that night, the patient returned to the emergency department because of a color change of the ear, worse along the superior helix, and increasing wound separation posteriorly (Fig 3, POD 14, 10). Although present earlier in the day, he was now noted to have an absent Doppler signal on the anterior surface of the ear. The vast majority of the ear remained well-perfused, likely from neovascularization at and around the lobule, which had the best skin-to-skin contact. The superior helix and the superior posterior skin were necrotic (Fig 6).

On POD 21, the patient was taken to the OR to debride the superior helix and postauricular skin necrosis. A superiorly based skin flap was created to bury the denuded helical cartilage, and the postauricular wound was closed directly (Fig 6). The remaining postoperative course was uneventful, and the patient was discharged home 3 days later.

**Figure 7:**
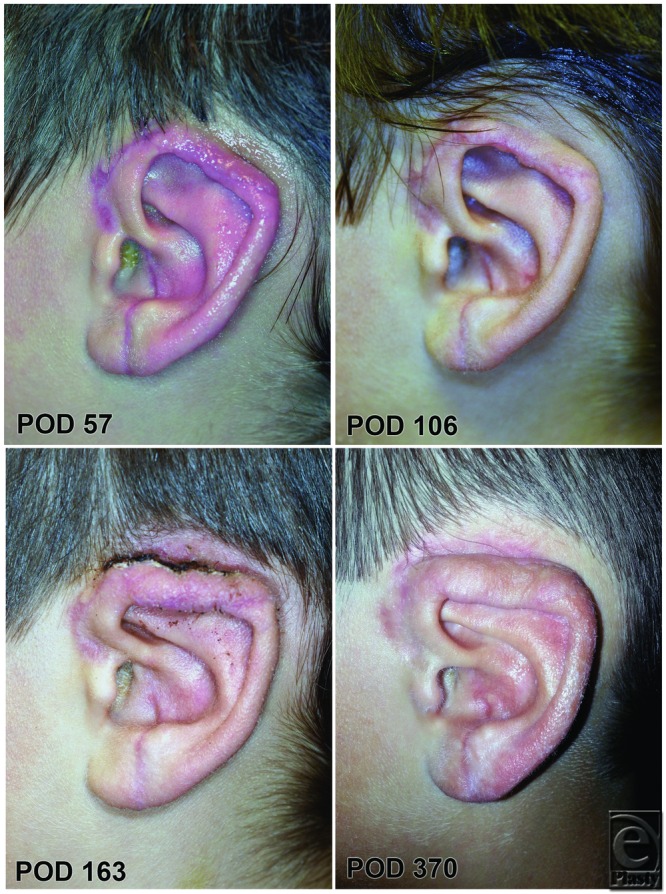
Long-term follow-up photographs of the replanted ear at various time points before full-thickness skin grafting to unbury the superior ear (top) and after (bottom). The patient and his family decided not to pursue further cartilage grafting to the superior helix for better contour. POD indicates postoperative day.

Five months later, the patient underwent release of the upper ear and full-thickness skin graft coverage of the remaining wound (Fig 7). The patient and his family elected not to pursue cartilage graft reconstruction of the upper ear (Fig 7, bottom right). One year postoperatively, the patient has regained sensation of the entire ear and notes only mild cold intolerance.

## DISCUSSION

Herein we report a successful subtotal ear replantation in a 10-year-old male child in which no suitable vein was encountered for repair. Extensive swelling and venous congestion were expected and successfully managed with leech therapy. Previous reports have described successful artery-only replantation, but none have documented the natural history of this treatment with photography.

While a consensus exists that microsurgical techniques vastly improve the outcome after ear replantation, the necessity of venous repair remains controversial.^2^ In general, replantation involves the establishment of arterial and venous anastomoses in addition to the physical reattachment of the amputated part. One of 2 arteries (posterior auricular artery or superficial temporal artery) is commonly used to revascularize the replanted ear. Either artery can provide sufficient perfusion to the entire ear.^3^ However, it is often difficult to find an adequately sized vein on the amputated part for venous anastomosis.

Multiple successful ear replants without venous anastomoses have been reported in the literature.^4^^-^18 These reports call into question the necessity of venous repair, given the risks of prolonged anesthesia, particularly if vein graft reconstruction is needed. One argument for venous repair is the restoration of the vascular circuit of the ear, thereby reducing postoperative venous congestion and bleeding.^19^ However, the classic study by Kind^20^ and the recent systematic review by Momeni et al^2^ demonstrate no significant difference in postoperative venous congestion or blood transfusions between artery-only replants and those with vein repair. Even with venous repair, there remains a high incidence of venous congestion and venous thrombosis, given the small diameter of involved veins and the relatively low-flow state within the vessel.^21^

If congestion is properly managed, natural venous outflow is typically established within 7 to 10 days,^4^^,^^9^^,^^22^^-^25 with natural arterial inflow following shortly thereafter. In this patient, the process of neovascularization could be clearly observed by day 12 starting at the lobule. The development of native arterial inflow and venous outflow was noted by progressive improvement in skin color progression from caudal to cephalad. Even with loss of the Doppler signal on day 14, the established neovascularization was sufficient to support the majority of the ear. Daily photography proved helpful in monitoring for subtle changes in neovascularization.

Medicinal leeches provide excellent relief of venous congestion following artery-only and arterial/venous ear replantation.^21^^,^^22^^,^^26^^,^^27^ Several factors released by leeches, such as anticoagulants, anesthetics, and vasodilators,^28^ make them the treatment of choice for venous congestion. Antibiotics such as ciprofloxacin, second- or third-generation cephalosporins, or trimethoprim/sulfamethoxazole can usually mitigate the risk of infection by *Aeromonas* species from leech therapy.^29^

In this case, full reperfusion of the ear was achieved for 4 days on the basis of a branch of the posterior auricular artery without a vein repair. However, arterial thrombotic complications led to an urgent take back to the OR, requiring vein graft reconstruction of the artery. This was likely due to compression of the repaired vessel from a postoperative hematoma caused by forceful vomiting in the setting of dual-agent anticoagulation. This complication undoubtedly contributed to compromise of the superior helical skin, but the majority of the ear was salvaged. The patient and his family were satisfied with the outcome 1 year postoperatively.

Arterial-only ear replantation is a viable solution when venous repair is not an option. Although venous repair should be attempted when possible, expending significant time to reconstruct venous outflow may not be warranted. Furthermore, the daily changes in appearance of an artery-only ear replantation have not previously been published. These images may serve as a useful tool for monitoring neovascularization or compromise of a replanted ear.

## Figures and Tables

**Table 1:** Leech schedule for artery-only subtotal ear replantation*

Postop day	Number of leeches	Leeching interval, hrs
0	2	2-3
3	1	4-6
4	Take back to OR, but anterior skin closure kept intact	
4, 0	2	1-2
6, 2	1	4
10, 6	1	6
12, 8	1	8
14, 10	Leech therapy discontinued	NA

*Leech therapy was used to maintain venous drainage of the replanted ear from postoperative days 0 to 14. They were increased temporarily after the take back to the operating room on day 4. Postop indicates postoperative; hrs, hours; OR, operating room; and NA, not applicable.
